# Low-Light Image Enhancement via Retinex-Style Decomposition of Denoised Deep Image Prior

**DOI:** 10.3390/s22155593

**Published:** 2022-07-26

**Authors:** Xianjie Gao, Mingliang Zhang, Jinming Luo

**Affiliations:** 1 Department of Basic Sciences, Shanxi Agricultural University, Jinzhong 030801, China; xjgao@sxau.edu.cn; 2 School of Mathematics and Statistics, Qilu University of Technology (Shandong Academy of Sciences), Jinan 250353, China; zhangml@qlu.edu.cn; 3 School of Mathematical Sciences, Dalian University of Technology, Dalian 116024, China

**Keywords:** low-light image enhancement, Retinex decomposition, Deep Image Prior

## Abstract

Low-light images are a common phenomenon when taking photos in low-light environments with inappropriate camera equipment, leading to shortcomings such as low contrast, color distortion, uneven brightness, and high loss of detail. These shortcomings are not only subjectively annoying but also affect the performance of many computer vision systems. Enhanced low-light images can be better applied to image recognition, object detection and image segmentation. This paper proposes a novel RetinexDIP method to enhance images. Noise is considered as a factor in image decomposition using deep learning generative strategies. The involvement of noise makes the image more real, weakens the coupling relationship between the three components, avoids overfitting, and improves generalization. Extensive experiments demonstrate that our method outperforms existing methods qualitatively and quantitatively.

## 1. Introduction

With the great breakthrough of deep learning in the field of computer vision technology, image processing has been widely used in many fields, e.g., face recognition [1], defect detection [2], medical image retrieval [3], traffic information systems [4], and text recognition [5]. Image defects can be attributed to uncontrolled factors such as insufficient lighting conditions and non-uniform lighting during image capture. These unfavorable elements can be disturbed by backlighting, underexposure, and night-time conditions. Low-light images are usually noisy, low-contrast, color-distorted, and quality-impaired. These shortcomings not only result in an unpleasant visual experience but also affect the performance of many computer vision systems, e.g., for image recognition, object detection, and image segmentation.

Image enhancement has a wide range of applications in different fields, e.g., underwater images [6], high-speed railway images [7], and robot vision [8]. In general, there are two ways to improve the image quality. One is to improve the hardware performance of photographic equipment and the other is to process the obtained image. However, the former has disadvantages such as manufacturing difficulties, high cost, and complicated technology. Therefore, in practical applications, improving the quality of low-light images through enhancement algorithms is of great significance. Low-light image enhancement has two main purposes: improving contrast and suppressing noise. The enhanced image is more suitable for human observation and computer vision systems.

Related studies on low-light image enhancement are reviewed, including those using conventional methods and deep learning methods. Traditional low-light enhancement methods include methods based on histogram equalization (HE) and the Retinex model. Histogram equalization is a method of using an image histogram to adjust contrast in the field of image processing (BPDHE [9], DHE [10], histogram modification [11]). HE methods may increase the contrast of noise and reduce the contrast of useful signals. In view of the shortcomings of the HE method, many improved versions have been proposed, e.g., clipped AHE [12], CLAHE [13], CVC [14], and contrast enhancement algorithm [15].

Retinex model-based methods decompose low-light images into reflection and illumination components [16]. Given a low-light image *S*, it can be decomposed into S=R⊙I, where *S* represents the low-light image, *R* represents the reflectance, *I* represents the illumination map, and ⊙ represents the dot product operation. In addition, many improved versions of Retinex models have been derived from the Retinex theory, including the single-scale Retinex model [17], the multi-scale Retinex model [18], the naturalness preserved enhancement algorithm [19], the fusion-based enhancing method [20], and illumination map estimation [21]. There are also some algorithms based on the variational Retinex model, e.g., the variational Retinex model formulated as a quadratic optimization problem [22], the variational framework for Retinex introducing a bright channel [23], the variational Retinex model based on the $L_{2}$-norm [24], the hybrid $L_{2}$-$L_{p}$ variational model with bright channel prior [25], and the maximum-entropy-based Retinex model [26]. Based on the computational complexity of variational methods, the disadvantage of this method is that processing images is time-consuming.

With the development of artificial intelligence, deep learning methods have also been widely used in the field of low-light image enhancement. Lore et al. [27] proposed a method of enhancing natural low-light images using a stacked sparse denoising autoencoder. Tao et al. [28] introduced a CNN method utilizing multi-scale feature maps to perform low-light image enhancement. Ignatov et al. [29] proposed a residual convolutional network that combines the composite perceptual error functions of content, color, and texture losses to improve the color and detail sharpness of the image. Shen et al. [30] put forward a convolutional neural network that directly learns the end-to-end mapping between dark and bright images for low-light image enhancement. Gharbi et al. [31] introduced a neural network architecture using input/output image pairs to perform image augmentation in real time and with full-resolution images. Wei et al. [32] designed a deep network called Retinex-Net based on the Retinex model, including Decom-Net for decomposition and Enhance-Net for lighting adjustment. Wang et al. [33] proposed a convolutional neural network based on the global prior information generated in the encoder–decoder network to enhance images. Chen et al. [34] presented a fully end-to-end convolutional network for processing low-light images using raw image data. Chen et al. [35] proposed an unpaired learning method for image enhancement based on a bidirectional generative adversarial network (GAN) framework. Zhang et al. [36] constructed an efficient network (KinD) trained on paired images shot under different exposure conditions. Wang et al. [37] proposed a neural network for enhancing underexposed photos by introducing intermediate lighting into the network to correlate the input with the expected enhancement result. Jiang et al. [38] proposed an unsupervised generative adversarial network trained with unpaired images. Yang et al. [39] suggested a semi-supervised learning method for low-light image enhancement based on a deep recursive band network (DRBN). Lv et al. [40] presented an end-to-end lightweight network for non-uniform illumination image enhancement that retains the advantages of the Retinex model and overcomes its limitations. Wang et al. [41] proposed the Deep Lightening Network (DLN) composed of several lightening back-projection (LBP) blocks to estimate residuals between low-light and normal-light images and the residual between low and normal light images. Zhu et al. [42] proposed the Edge-Enhanced Multi-Exposure Fusion Network (EEMEFN), which includes a multi-exposure fusion module and an edge enhancement module to enhance extremely low-light images. Liu et al. [43] obtained a Retinex-inspired Unrolling with Architecture Search (RUAS), where a cooperative architecture search was used to discover low-light prior architectures from a compact search space, and reference-free losses were used to train the network. Li et al. [44] presented a progressive–recursive image enhancement network (PRIEN) that uses a recursive unit to progressively enhance the input image. Zhang et al. [45] proposed dynamic fields to learn and make inferences from a single image, and then enforce temporal consistency. Fu et al. [46] suggested a novel unsupervised low-light image enhancement network (LE-GAN) based on generative adversarial networks using unpaired low-light/normal-light images for training. Zhao et al. [47] proposed a unified deep zero-reference framework termed RetinexDIP for enhancing low-light images; however, noise was not considered in the decomposition process. Liu et al. [48] proposed the Retinex-based fast algorithm (RBFA) to achieve low-light image enhancement. Liang et al. [49] proposed a low-light image enhancement model based on deep learning. Li et al. [50] presented a low-light image enhancement method based on a deep symmetric encoder–decoder convolutional network. Han et al. [51] proposed a DIP based on a noise-robust super resolution method. Ai and Kwon [52] used attention U-Net for extreme low-light image enhancement. Zhao et al. [53] proposed a multi-path interaction network to improve the quality of the image.

In this paper, we propose a novel RetinexDIP method to enhance images. Noise components are introduced into our network, and three components are generated by the DIP network. The involvement of noise makes the image more real, weakens the coupling relationship between the three components, avoids overfitting, and improves generalization. The illumination map is obtained by iterating and adjusting the input noise, and then the enhanced image is generated based on the Retinex model. Our training process is a zero-reference process and does not require any paired or even unpaired data, which is similar to existing methods (EnlightenGAN [38], CycleGAN [54], Zero-DCE [55]). The novel RetinexDIP method can be applied to various poorly lit environments and has good generalization. The loss function in this paper is composed of four parts: the spatial reconstruction loss, illumination-consistency loss, reflectance loss, and illumination smoothness loss. The experimental results show that the normal light images generated by our method are natural and clear and the method has excellent performance according to both visual observation and objective evaluation indicators. The main contributions of this paper are as follows:

1. We propose a novel noise-added RetinexDIP method to enhance images.
2. Three components are generated by the DIP network.
3. The zero-reference process avoids the risk of overfitting and improves generalization.
4. The experimental results show that our method significantly outperforms some current state-of-the-art methods.

The rest of the paper is organized as follows. Section 2 details our proposed approach. Section 3 presents the experimental results, and the last section concludes the paper.

## 2. Materials and Methods

Given a low-light image *S* and considering noise, the image can be decomposed into:

(1) S=R⊙I+N

or

(2) S=(R+N)⊙I,

where *S* represents the low-light image, *R* represents the reflectance, *I* represents the illumination map, *N* denotes the noise, and ⊙ represents the dot product operation. Adding hand-crafted priors to components makes the components more coupled. Deep Image Prior (DIP) means that complex prior knowledge does not need to be introduced, as it can be encoded in the structure of the neural network itself  [56]. In practical problems, it is difficult to find pairs of low-light and normal images. Therefore, generative models are becoming more and more important.

**Figure 1:**
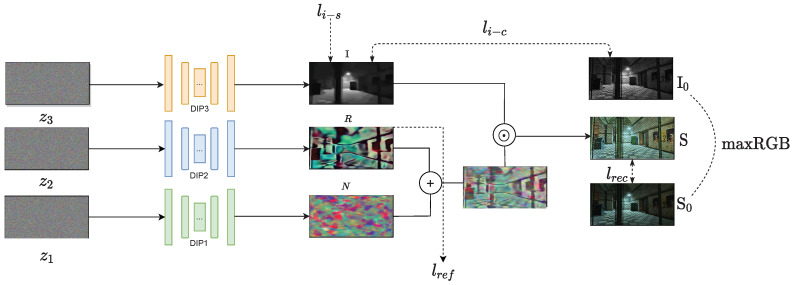
Overall framework of our method, where $z_{1,2,3}∼N\left(0,\sigma^{2}\right)$ signify Gaussian noise. DIP1 is used to generate noise *N*, and DIP2 and DIP3 are used to generate the reflectance *R* and the latent illumination *I*. $l_{rec}$, $l_{i-c}$, $l_{ref}$, and $l_{i-s}$ represent reconstruction loss, illumination-consistency loss, reflectance loss, and illumination smoothness loss, respectively. $I_{0}$ is the initial illumination, $S_{0}$ is the input image, and *S* is the enhanced image.

In this paper, we implement image decomposition based on Retinex theory and generative strategies, taking into account the noise factor. The overall framework of this method is shown in Figure 1. As can be seen from Figure 1, there are three encoder–decoder networks (DIP1, DIP2, and DIP3) in the model. These DIP networks are all convolutional operations. DIP1 is used to generate noise *N*, and DIP2 and DIP3 are used to generate the reflectance *R* and the latent illumination *I*. All three DIP networks use white Gaussian noise as input and obey $z_{1,2,3}∼N\left(0,\sigma^{2}\right)$, where $\sigma^{2}$ represents the variance of the Gaussian distribution. The noise is obtained via random sampling and has the same size as the image.

To evaluate the quality of the augmented images, the following four types of losses were employed to train our model.

**Reconstruction Loss.** The reconstruction loss is defined according to the following form:

(3) $$l_{rec}={∥g_{I}\left(z_{3}\right)⊙\left(g_{R}\left(z_{2}\right)+g_{N}\left(z_{1}\right)\right)-S_{0}∥}_{2}^{2},$$

where *N* is the noise generated by DIP1, denoted by $g_{N}$, *R* is the latent reflectance generated by DIP2, denoted by $g_{R}$, and *I* is the illumination generated by DIP3, denoted by $g_{I}$. $S_{0}$ is the observed image.

**Illumination-consistency Loss.** As in [47], we also consider the illumination-consistency loss, which is defined as

(4) $$l_{i-c}={∥g_{I}\left(z_{3}\right)-I_{0}∥}_{1},$$

where $I_{0}$ is the initial illumination obtained by

(5) $$I_{0}\left(p\right)=\max_{c\in \{R,G,B\}}S_{c}\left(p\right)$$

for every pixel *p*.

**Reflectance Loss.** In this paper, the reflectance *R* is considered, and the total variation (TV) constraint [57] is defined as

(6) $$l_{ref}={∥\nabla g_{R}\left(z_{2}\right)∥}_{1},$$

where ∇ denotes the first-order operator containing a horizontal component $\nabla_{h}$ and a vertical component $\nabla_{v}$.

**Illumination Smoothness Loss.** We also use the illumination reflection gradient-weighted TV constraint, defined as

(7) $$l_{i-s}={∥W⊙\nabla g_{I}\left(z_{3}\right)∥}_{1},$$

where *W* is the weight matrix. According to the weight strategy in [21], it is set via:

(8) $$W_{h,v}\leftarrow \frac{1}{|\nabla_{h,v}I_{0}|+\epsilon },$$

where ϵ is a small decimal to ensure that the denominator is not 0.

Combining the four losses, we minimize the objective function as follows:

(9) $$\arg\min_{I,R,N}∥g_{I}\left(z_{3}\right)⊙\left(g_{R}\left(z_{2}\right)+g_{N}\left(z_{1}\right)\right)-S_{0}{∥}_{2}^{2}+\lambda_{1}∥g_{I}\left(z_{3}\right)-I_{0}{∥}_{1}+\lambda_{2}∥\nabla g_{R}\left(z_{2}\right){∥}_{1}+\lambda_{3}{∥W⊙\nabla g_{I}\left(z_{3}\right)∥}_{1},$$

where $\lambda_{1}$, $\lambda_{2}$, and $\lambda_{3}$ are the balance parameters.

The enhanced image *S* is composed of noise *N*, reflectance *R*, and the latent illumination *I*.

(10) S=(R+N)⊙I,

or

(11) $$S=g_{I}\left(z_{3}\right)⊙\left(g_{R}\left(z_{2}\right)+g_{N}\left(z_{1}\right)\right).$$

Next, enhancement using only estimated illumination is described. There are two commonly used composition strategies: one is to remove the illumination component, considering the reflectance as the enhancement result, i.e.  $\hat{S}=S/I$, and the other is to adjust the illumination and reconstruct the result with the reflectance, i.e.  $\hat{S}=\hat{I}⊙R$. In this paper, we use a variant of the former strategy, that is, $\hat{S}=S/\hat{I}$ (refer to [21] for details).

We adjust the illumination distribution of decomposition using the gamma correction $\hat{I}=I^{\gamma }$, where γ is the correction factor. To sum up, the enhanced result is given by:

(12) $$\hat{S}=S^{c}/\hat{I},\;c\in \left\{R,G,B\right\}.$$

The whole operation process is shown in Algorithm 1.
**Algorithm 1:** our algorithm
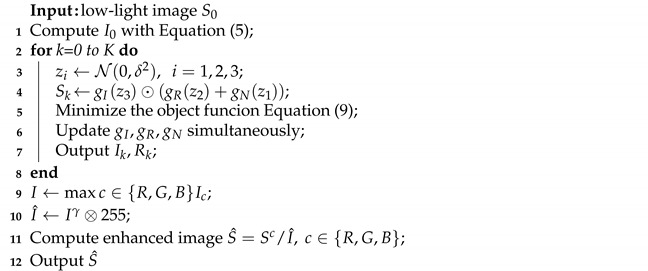


## 3. Experiment

In this section, the experimental parameter settings, public low-light image datasets, and performance metrics are introduced. The results of our approach with different methods are also be discussed.

### 3.1. Settings

We implement our framework using PyTorch on an NVIDIA 2080Ti GPU. The model experimental parameters were set as follows: $\lambda_{1}=1$, $\lambda_{2}=0.0001$, $\lambda_{3}=0.5$, δ=0.01, γ=0.5, and K=300. We use six public datasets with low-light images for the experiments, including DICM [15], Fusion [58], LIME [21], MEF [59], NPE [19], and VV (https://sites.google.com/site/vonikakis/datasets (accessed on 1 June 2022)).

### 3.2. Performance Criteria

In this paper, we measure the experimental results from visual observations and objective evaluation indicators. The following evaluation indicators were used.

**Natural Image Quality Evaluator (NIQE).** The inspiration for NIQE is based on constructing a series of features used to measure image quality and using these features to fit a multivariate Gaussian model. In the evaluation process, the distance between the image feature model parameters (to be evaluated) and the pre-established model parameters is used to determine the image quality. A lower NIQE score indicates better preservation of naturalness. For details, refer to [60].

**No-reference Image Quality Metric for Contrast Distortion (NIQMC).** NIQMC is defined as a simple linear fusion of global and local quality measures [61]. A higher NIQMC score represents better image contrast.

**Colorfulness-Based Patch-Based Contrast Quality Index (CPCQI).** CPCQI is a color-based PCQI metric that evaluates the enhancement effect between input and enhanced output in terms of mean strength, signal strength, and signal structure components [62]. A larger CPCQI value indicates a higher contrast ratio.

### 3.3. Results

In this section, we show the effectiveness of the proposed method. We compare it with six other methods, i.e., LIME [21], NPE [19], SRIE [63], KinD [36], Zero-DCE [55], and RetinexDIP [47].

**Figure 2:**
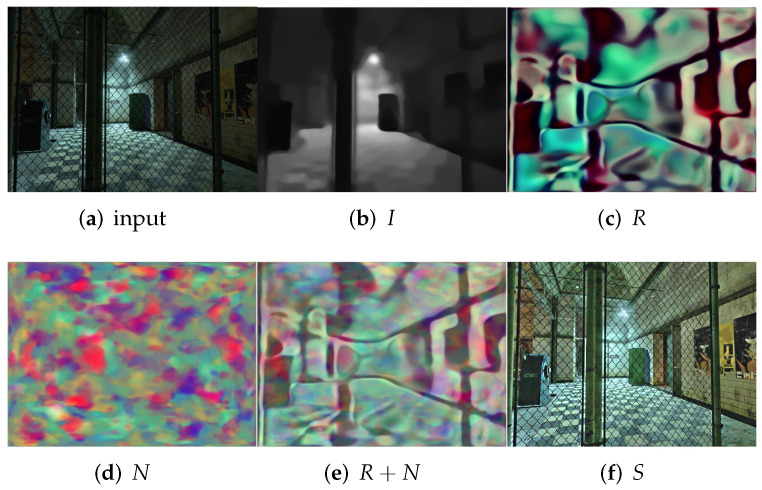
Results of each step of our method: (**a**,**f**) are the input image and enhanced result; (**b**–**e**) represent illumination *I*, reflectance *R*, noise *N*, and R+N.

The specific process for our method is shown in detail step by step in Figure 2.

**Figure 3:**
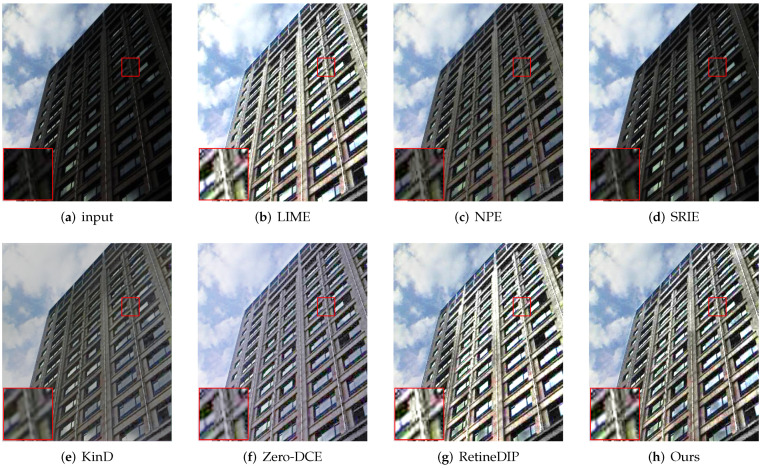
Comparisons of enhanced images. Red boxes indicate the obvious differences. Compared with other methods, our method yields natural exposure and clear details.

**Figure 4:**
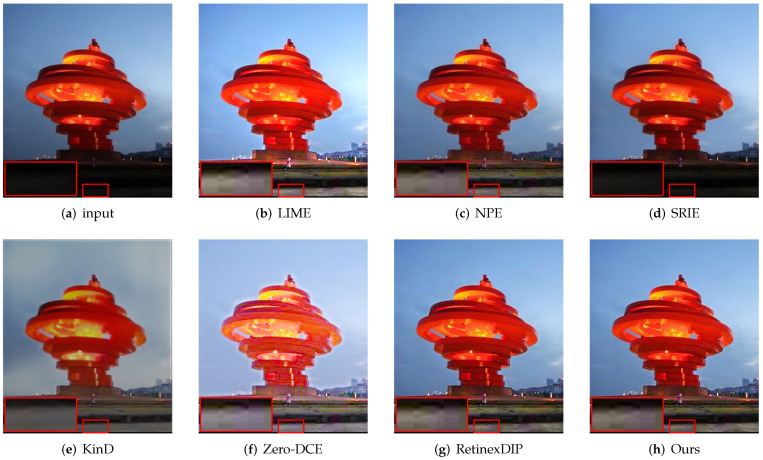
Comparisons of enhanced images. Red boxes indicate the obvious differences. Compared with other methods, our method enhances the image and the edges are clearly visible.

**Figure 5:**
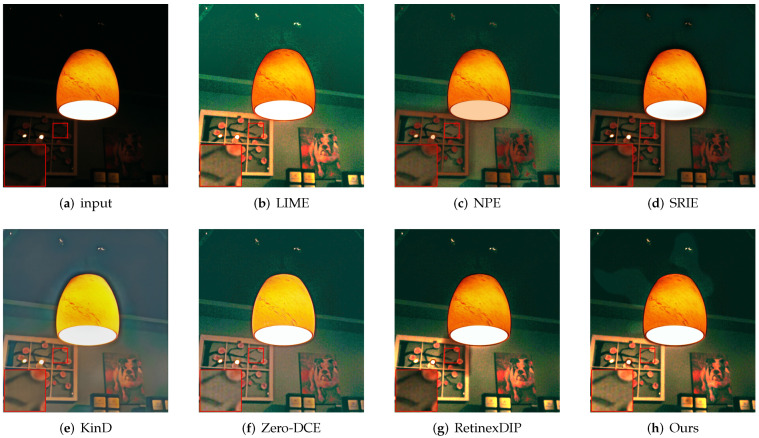
Comparisons of enhanced images. Red boxes indicate the obvious differences. Compared with other methods, our method does not have the problems of overexposure and artifacts.

**Figure 6:**
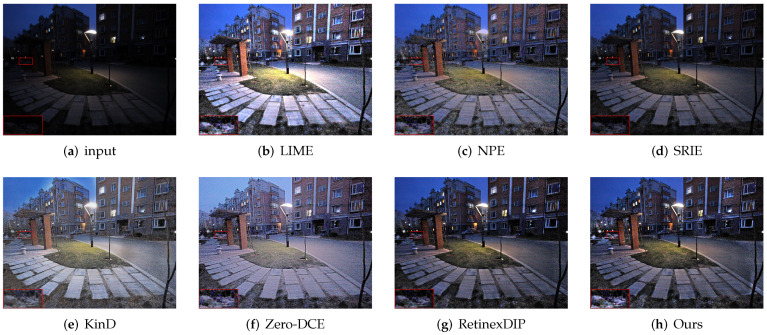
Comparisons of enhanced images. Red boxes indicate the obvious differences. Compared with other methods, our method improves the contrast effectively and maintains the natural color.

First, we evaluate the different methods qualitatively. As shown in Figure 3, Figure 4, Figure 5 and Figure 6, we select local regions and zoom in on them for intuitive comparison with other methods. The following conclusions can be drawn from the observation of Figure 3. The enhancement effect of the NPE, SRIE, and KinD methods is not obvious. The LIME and RetinexDIP methods produce over-enhancement effects in these regions. The processing result of Zero-DCE has unnatural color. Our method yields natural exposure and clear details. In Figure 4, it can be seen that our method enhances the image and the edges are clearly visible. The result of KinD has an unnatural color. By considering Figure 5, it can be seen that the method proposed in this paper does not have the problems of overexposure and artifacts when improving the contrast. From Figure 6, it can also be concluded that our method improves the contrast effectively and maintains the natural color at the same time.

In the following, we compare the proposed method with other methods quantitatively. The red, green, and blue scores represent the top three in the corresponding dataset, respectively. Table 1 presents the NIQE metrics of different methods on the six datasets. Notably, a lower NIQE score indicates better preservation of naturalness. Our method achieves the best results on the MEF and VV datasets and the second-best results on the average of the six datasets and LIME. Table 2 presents the NIQMC metrics of the different methods on the six datasets. A higher NIQMC score represents better image contrast. Our method is in the top three for DICM, LIME, MEF, NPE, VV, and the average of the six datasets. Table 3 presents the CPCQI of the different methods on the six datasets. A larger CPCQI value indicates a higher contrast ratio. Our method achieves the best results on DICM, Fusion, NPE, and the average of the six datasets, and also performs well on several other datasets.

As shown in Table 4, the runtimes of different methods were compared. In the experiment, we compare the runtimes of three traditional methods (LIME, NPE, SRIE) and three deep learning methods (KinD, Zero-DCE, RetinexDIP) to that of our method, with eight different input image sizes. Compared with NPE, SRIE, and RetinexDIP, we find that our method is more efficient on high-resolution images. Unlike traditional methods such as NPE and SRIE, the proposed method uses the DIP network to compute reflections and illumination. Benefiting from the convolutional structure, the runtime of the DIP model changes very little as the image resolution grows. Compared with RetinexDIP, the proposed method converges faster and requires less runtime due to the consideration of noise. Compared with Zero-DCE and KinD, our method can also save memory, since Zero-DCE and KinD are pixel-wise methods, while the proposed method is based on Retinex decomposition. The proposed method does not require the actual resolution of the image in the operation, and the memory will not increase significantly with an increase in the image resolution.

## 4. Conclusions

In this paper, we propose a novel low-light image enhancement method via Retinex decomposition of denoised Deep Image Prior. Noise is considered in the image decomposition using deep learning generative strategies. As a comparison, we also consider six other methods, i.e., LIME, NPE, SRIE, KinD, Zero-DCE, and RetinexDIP. Extensive experiments demonstrate that our method outperforms existing methods qualitatively and quantitatively. Unlike some other learning-based methods, the method proposed in this paper is a no-reference method, which means that only the input images are required without any extra data. Taking the reflection noise into consideration, our experiments show that the denoised Deep Image Prior can produce images with less noise.

In real scenes, noise always conforms to some scene-dependent distribution such as the Poisson distribution. In future work, other approaches such as normalizing flow will be considered to simulate a more realistic noise distribution than that of DIP.

## Figures and Tables

**Table 1:** Comparison of average NIQE on six datasets. (The red, green, and blue scores represent the top three in the corresponding dataset, respectively).

Method	DICM	Fusion	LIME	MEF	NPE	VV	Average
LIME	3.5360	3.9183	4.1423	3.7022	4.2625	2.7475	3.5442
NPE	3.4530	3.8883	3.9031	3.5155	3.9501	3.0290	3.4928
SRIE	3.5768	3.9741	3.7868	3.4742	3.9883	3.1357	3.5668
KinD	4.2691	4.1027	4.3525	4.1318	3.9589	3.4255	4.0752
Zero-DCE	3.6091	4.2421	3.9354	3.4044	4.0944	3.2245	3.6332
RetinexDIP	3.7612	4.2308	3.6355	3.2721	4.1012	2.4890	3.5363
Ours	3.7911	4.0628	3.7615	3.2363	4.0426	2.4604	3.5294

**Table 2:** Comparison of average NIQMC on six datasets. (The red, green, and blue scores represent the top three in the corresponding dataset, respectively).

Method	DICM	Fusion	LIME	MEF	NPE	VV	Average
LIME	5.3397	5.3686	5.4956	5.4168	5.4480	5.5805	5.4121
NPE	5.0895	4.5802	4.6168	4.8610	5.1738	5.2655	5.0104
SRIE	4.9990	4.3568	4.5032	4.7045	5.1848	5.3021	4.9246
KinD	4.6155	4.5248	4.6841	4.6725	4.5766	4.8159	4.6511
Zero-DCE	4.8984	4.7346	5.0678	5.0504	5.1068	5.3614	5.0062
RetinexDIP	4.9912	4.4449	4.7830	5.0151	5.3222	5.3915	5.0126
Ours	5.0093	4.5210	4.7996	5.0761	5.2931	5.4138	5.0398

**Table 3:** Comparison of average CPCQI on six datasets. (The red, green, and blue scores represent the top three in the corresponding dataset, respectively).

Method	DICM	Fusion	LIME	MEF	NPE	VV	Average
LIME	0.8986	0.9642	1.0882	1.0385	0.9844	0.9555	0.9515
NPE	0.9139	0.9705	1.0812	1.0372	1.0228	0.9557	0.9609
SRIE	0.9056	1.0094	1.1121	1.0967	1.0258	0.9629	0.9721
KinD	0.7459	0.8148	0.8336	0.7877	0.8007	0.7418	0.7670
Zero-DCE	0.7818	0.8820	0.9803	0.9461	0.8578	0.8396	0.8415
RetinexDIP	0.9999	1.0680	1.1595	1.1088	1.0411	1.0525	1.0436
Ours	1.0038	1.0787	1.1585	1.0926	1.0524	1.0445	1.0437

**Table 4:** Runtime (RT) comparison (in seconds).

Method	(640×480)	(1280×960)	(1920×1440)	(2560×1920)	(3200×2400)	(3840×2880)	(4480×3360)	(5120×3840)
LIME	0.1133	0.4196	1.0148	1.5713	2.3901	3.3302	4.4058	5.7054
NPE	5.8861	26.6340	58.5019	104.8345	163.9938	235.7513	326.0996	427.1531
SRIE	4.7643	33.6684	121.5802	343.9839	726.5981	386.7066	544.0660	865.0404
KinD	0.1554	0.0464	-	-	-	-	-	-
Zero-DCE	0.12559	0.1390	0.2539	0.4051	0.83371	-	-	-
RetinexDIP	15.2482	15.5945	31.4575	52.1564	102.5126	139.4568	182.4861	212.1594
Ours	15.3655	15.4910	30.8131	48.9527	94.5498	122.6732	154.7097	189.1483

## Data Availability

Not applicable.
